# Association of ideal cardiovascular health with cardiovascular events and risk advancement periods in a Mediterranean population-based cohort

**DOI:** 10.1186/s12916-022-02417-x

**Published:** 2022-07-05

**Authors:** Cesar I. Fernandez-Lazaro, Carmen Sayon-Orea, Estefania Toledo, Conchi Moreno-Iribas, María J. Guembe, for the RIVANA Study Investigators, Joaquín Barba Cosials, Jesús Berjón Reyero, Javier Díez Martínez, Paulino González Diego, Ana Mª. Grijalba Uche, David Guerrero Setas, Eduardo Martínez Vila, Manuel Serrano Martínez, Isabel Sobejano Tornos, José Javier Viñes Rueda

**Affiliations:** 1 Department of Health, Government of Navarre, Vascular Risk in Navarre Investigation Group, Pamplona, Spain; 2 Department of Preventive Medicine and Public Health, School of Medicine, University of Navarra, C/Irunlarrea 1, 31008 Pamplona, Spain; 3 IdiSNA, Navarra Institute for Health Research, Pamplona, Spain; 4 Navarrabiomed-Miguel Servet Foundation, Pamplona, Spain; 5 Navarra Public Health Institute, Pamplona, Spain; 6 CIBER of Epidemiology and Public Health (CIBERESP), Madrid, Spain; 7 Health Services Research on Chronic Patients Network (REDISSEC), Institute of Health Carlos III, Madrid, Spain; 8 Servicio de Apoyo a la Gestión Clínica y Continuidad Asistencial, Complejo Hospitalario de Navarra, Pamplona, Spain; 9 Dirección General de Salud del Gobierno de Navarra, Servicio de Planificación, Evaluación Y Gestión del Cono-cimiento, Pamplona, Spain

**Keywords:** Ideal cardiovascular health, Life’s Simple 7, Cardiovascular risk, Rate advancement periods, RIVANA study

## Abstract

**Background:**

The American Heart Association recommends Life’s Simple 7 as ideal cardiovascular health (ICVH) to reduce cardiovascular risk. Rate advancement period (RAP), a useful tool to quantify and communicate exposure impact on risks, may enhance communication about the benefits of achieving ICVH. We aimed to examine whether greater adherence to ICVH metrics was associated with reduced incidence of cardiovascular risk in a population-based cohort and estimate its impact on the timing of occurrence using RAP.

**Methods:**

Prospective analyses of 3826 participants, initially free from cardiovascular disease at baseline, enrolled in the Vascular Risk in Navarra Study (RIVANA), a Mediterranean population-based cohort of Spanish adults. ICVH metrics were defined using participants’ baseline information as follows: never-smoker or quitting > 12 months ago, body mass index < 25 kg/m^2^, ≥ 150 min/week of moderate physical activity or equivalent, healthy dietary pattern (≥ 9 points on a validated 14-item Mediterranean diet adherence screener), untreated cholesterol < 200 mg/dL, untreated blood pressure < 120/80 mmHg, and untreated fasting blood glucose < 100 mg/dL. Participants were assigned 1 point for each achieved metric and were grouped according to their number of accumulated metrics in ≤ 2, 3, 4, and ≥ 5. The primary endpoint was major cardiovascular events (composite of myocardial infarction, stroke, or death from cardiovascular causes). Cox proportional hazard ratios (HRs) and RAPs with their corresponding 95% confidence intervals (95% CI) adjusted for potential confounders were calculated.

**Results:**

During a median follow-up of 12.8 years (interquartile range 12.3–13.1), a total of 194 primary endpoints were identified. Compared to participants with ≤ 2 ideal metrics, HR (95% CI) for major cardiovascular events among participants meeting ≥ 5 metrics was 0.32 (0.17–0.60) with RAP (95% CI) of − 14.4 years (− 22.9, − 5.9).

**Conclusions:**

Greater adherence to ICVH metrics was associated with lower cardiovascular risk among Spanish adults of the RIVANA cohort. Adherence to ideal metrics may substantially delay cardiovascular risk.

**Supplementary Information:**

The online version contains supplementary material available at 10.1186/s12916-022-02417-x.

## Background

Cardiovascular disease (CVD) is the leading cause of death globally. Approximately 18.6 million people died worldwide from CVDs in 2019, and global trend predictions suggest that the number of deaths due to CVD will continue growing [1]. In the USA, one person currently dies from CVD every 36 s [2], and in Europe, 3.9 million deaths occur each year [3]. Modifiable risk factors are responsible for over 70% of cardiovascular cases and deaths [4], and a large fraction of cardiovascular deaths may be preventable by adhering to certain potentially modifiable dietary and lifestyle healthy habits [5].

In 2010, the American Heart Association (AHA) released the 2020 Impact Goals aiming to improve overall health and reduce deaths from CVD. For this purpose, the AHA introduced the concept of ideal cardiovascular health (ICVH), based on 7 metrics (Life’s Simple 7) [6], that included 4 ideal health behaviors (healthy diet, non-smoking, appropriate physical activity levels, and body mass index [BMI]) and 3 ideal health factors (optimal levels for untreated total cholesterol, untreated blood pressure, and untreated fasting blood glucose). Since then, several studies have been conducted across different countries supporting the benefits of adhering to these 7 metrics [7, 8]. Despite that, a low prevalence of ICVH metrics has been observed globally [9].

Effective risk communication is essential to enhance public understanding of the positive effects of adhering to certain health factors and may help individuals to change their behavior [10, 11]. However, risk messages can be frequently challenging to understand for the general population, particularly if they are expressed with classical risk estimates [12]. In this context, rate advancement period (RAP), proposed by Brenner et al. [13], is an epidemiologic measure that expresses the impact of a certain risk factor in terms of time (years of chronological age) of chronic disease occurrence or related to mortality, in the absence of competing risks. In this sense, RAP may facilitate the cardiovascular risk interpretation by quantifying the beneficial effects of achieving ICVH metrics in terms of premature or delayed disease risk, similar to the concept of years of life lost [14, 15]. RAP has been previously reported to enhance risk communication on the positive health effects of quitting smoking [16], achieving healthy lifestyle behaviors [17], and adhering to dietary quality patterns [18]. However, the use of RAP is not widely used in epidemiological studies.

In Spain, only one prospective study has examined the association between ICVH metrics and major cardiovascular events [19]. This research included an elderly population at high cardiovascular risk who received an intense behavioral dietary educational intervention within the frame of the PREDIMED, a parallel-group, multicenter, randomized trial [20, 21]. However, no population-based study has examined the association between ICVH metrics and CVD in Spain. Moreover, to our knowledge, no study has yet used RAPs to quantify years of life that can be potentially gained when achieving ICVH. We aimed to prospectively investigate whether the ICVH metrics were associated with lower cardiovascular events in a population-based Mediterranean cohort over 13 years. We additionally aimed to ascertain the effect of ICVH metrics on the relation of age to the risk of cardiovascular events occurrence using RAPs.

## Methods

The present study is embedded in the Vascular Risk in Navarra (RIVANA) Project, a Mediterranean population-based cohort designed to assess the prevalence of cardiovascular risk factors and their association with CVD morbidity and mortality. Details of the study design and procedures have been described elsewhere [22, 23]. Briefly, a total sample of 6553 individuals aged 35–84 years was randomly selected from the population register of the Autonomous Community of Navarre, Spain. The sample was stratified by sex and age to be representative of the Navarre population. Individuals who were institutionalized, living in Autonomous Communities other than Navarre, had died, and did not respond after multiple contacts were excluded from the initial sample. The logistics of the study implied the deliberate exclusion of institutionalized individuals and people living temporarily in Autonomous Communities other than Navarre when the study was carried out [22]. After inclusion/exclusion criteria, 5682 individuals were invited through mail, phone calls, and home visits from June 2004 to December 2005 to participate in the study cohort. Of these, 4168 individuals agreed to enroll in the cohort (response rate 73.4%) and were followed up until December 2017.

The study protocol was approved by The Institutional Review Board of the Government of Navarre (approval code PI_2004/4). All participants provided written informed consent to participate in the study and additional access to their medical records.

For the present analyses, we excluded participants with prevalent CVD (ischemic heart disease, cerebrovascular disease, or peripheral arterial disease) at baseline (*n* = 333), those with missing values for health metrics (*n* = 4), and those who were lost to follow-up (*n* = 5), with an overall retention > 99%.

### Assessment of the cardiovascular health score and covariates

Participants that agreed to enroll in the cohort were contacted from one of the 25 participating primary healthcare centers. Trained nurses collected via face-to-face structured interviews participants’ self-reported socio-demographic and lifestyle information, including smoking habit, physical activity, adherence to the Mediterranean diet (MedDiet), individual and family medical history, and current medication use. The pharmacist and physician team members reviewed the participants’ medication for each condition and resolved with nurses any potential misclassification or conflict. Adherence to the MedDiet was measured using a previously validated 14-item questionnaire [24, 25]. Physical activity levels were assessed using the validated Spanish version of the Minnesota Leisure Time Physical Activity Questionnaire [26, 27]. Metabolic equivalent hours per week (METs-h/week) were calculated based on time spent and intensity required for each performed activity according to the international compendium of physical activity [28]. Anthropometric measures were conducted in duplicate by registered nurses using standardized techniques. Blood pressure was measured in triplicate using a validated semiautomatic oscillometer (OMRON® M4-1). Finally, laboratory test analyses were conducted to determine biological parameters, including fasting blood glucose, lipid profile, and high-sensitivity C-reactive protein level, among others [22, 23].

Our ICVH score was based on 7 equally weighted health metrics as proposed by the AHA [6]. We assigned 1 point for each of the following health metrics that participants met at baseline: never-smoker or quitting > 12 months ago, ≥ 9 points of adherence to MedDiet, ≥ 150 min/week of moderate physical activity or ≥ 75 min/week of vigorous intensity or an equivalent combination, BMI < 25 kg/m^2^, untreated total cholesterol < 200 mg/dl, untreated blood pressure < 120/80 mmHg, and untreated fasting glucose < 100 mg/dl. Physical activity at “goal” was defined based on the World Health Organization recommendations [29], which is considered to be the equivalent of ≥ 500 METs-min/week based on the Compendium of Physical Activities (using a value of 3.3 METs, the level associated with a brisk [3 miles per hour] walking pace) [30]. This ≥ 500 METs-min/week cut-off was also used in previous publications to define physical activity guidelines [19, 31]. The MedDiet was selected as dietary pattern that ‘promotes cardiovascular health’ due to the large, robust, and consistent evidence of its cardiovascular beneficial effects [32, 33]. The ≥ 9 cut-point score was selected based on the definition of good adherence provided by previous MedDiet studies [34, 35]. Thus, the total ICVH score ranged from 0 (worst) to 7 (best) points. Participants were categorized into 4 groups according to the number of achieved metrics (0–2, 3, 4, and 5–7) to ensure an adequate distribution of the sample in each score group. The upper and lower extremes of the ICVH score range were collapsed due to the low proportion of participants with these numbers of metrics.

### Outcome assessment

The primary endpoint of the study was a composite of myocardial infarction, stroke, or death from cardiovascular causes  (all major cardiovascular events). The secondary endpoints were the individual components of the main composite endpoint and an expanded composite of major cardiovascular events that additionally included other ischemic heart diseases, other cerebrovascular diseases, and peripheral arterial disease. Definitions of study endpoints are described in Additional file 1: Fig. S1. For the ascertainment of cardiovascular events, several sources of information were used: primary electronic health record database, hospital discharge database, population based myocardial infarction registry, and regional and national mortality registries (managed by the National Statistical Institute, Madrid, Spain), which provided information on date and cause of death. Nearly all the 650,000 inhabitants of the community of Navarre are covered by the primary care electronic health record database, which has resulted to be a valid source for epidemiological surveillance in previous validation studies of disease diagnoses [36, 37]. To further identify potential cardiovascular events requiring hospitalization, we additionally reviewed the Assisted Morbidity Registry of Navarre, which involved the Minimum Basic Data Set with information of all public and private hospitals in the Navarre Health System. Record linkage additionally included the Regional Registry of Myocardial Infarction of Navarre and the National Statistics Institute of Spain. The combination of 4 sources of information and the utilization of primary electronic health records assured the study to have a strict follow-up of vital status of participants (< 1% losses). Medical records were reviewed by members of the study team. A validation process was carried out to confirm and classify the cardiovascular events identified in the different information sources. Detailed information of the outcome assessment can be found somewhere else [23].

### Statistical analysis

Descriptive statistics were used to summarize the distribution of the baseline characteristics of the study population across groups of the ICVH.

We used Nelson-Aalen estimators, adjusted for potential confounders with inverse probability weighting [38], to describe the incidence of the study’s endpoints over follow-up time across the 4 groups, and the differences between the groups were tested using the log-rank test.

Multivariate Cox proportional hazards models were used to calculate the hazard ratios (HRs) and 95% confidence intervals (CIs) of risk of developing cardiovascular events during follow-up. We considered the lowest score group (0–2 metrics) as the reference category. Time on study was the interval between the date of completion of the baseline interview and the date of diagnosis of cardiovascular events, or the date of death, whichever occurred first and the last recorded follow-up for non-cases. Follow-up of participants without cardiovascular events exceeding the end of follow-up date (31 December 2017) was censored at this date. After crude models, we fitted age and sex-adjusted models, and multivariable models additionally adjusted for higher level of attained education (primary or less, secondary, and college/university), and occupation (executives/managers, clerical workers, and manual workers).

Tests of linear trend across successive categories of the ICVH score were calculated by assigning the median value to each category treating the resulting variables as continuous. The potential non-linear relationship between ICVH metrics and the primary endpoint was graphically examined using fully adjusted restricted cubic splines with three knots. A *p*-value for non-linearity was calculated by the likelihood ratio test [39].

RAPs for the study endpoints were calculated according to Brenner et al. [13]. In our study, RAPs represent the equivalent risk associated with ICVH metric in terms of years of chronological age. Thus, RAPs represent the baseline age difference at which exposed subjects (participants with > 2 metrics) reach the same risk of disease (endpoint events) as unexposed subjects (participants with ≤ 2 metrics). Point estimates of RAPs and 95% CIs were derived from multivariate Cox proportional hazard models adjusted for the same potential confounders. We verified the proportionality of hazard assumption graphically and conducting Schoenfeld residuals to correctly interpret RAP estimates [14, 40].

The association of each individual health metric with the risk of developing major cardiovascular events was additionally calculated. Cox models were fitted for each health metric of the score adjusting the models for the effect of the other metrics and the same confounders as previous models. The reference category for each independent analysis was not meeting the criterion (0 points).

Sensitivity analyses were also conducted using additional adjustments: alcohol consumption (never [0 g/day]; light [< 5 g/day]; moderate [women: 5–15 g/day and men 5–30 g/day]; heavy [women: > 15 g/day and men > 30 g/day]), and serum concentration of high-sensitivity C-reactive protein (continuous). We additionally explored the association between individual health metrics and the primary endpoint of the study by removing from the models the adjustments of the effect of the other metrics.

All statistical tests were conducted using STATA version 16 (StataCorp LP), and statistical significance was set at 2-tailed *p* < .05.

## Results

A total of 3826 participants (mean [SD] age, 52.8 [12.8] years; 2137 [55.9%] female) were included for the present analyses. During a median of 12.8 years (interquartile range 12.3 to 13.1) of follow-up, a total of 194 primary endpoints (major cardiovascular events) were identified. For secondary endpoints, we identified 67 myocardial infarction events, 87 stroke events, 66 deaths from cardiovascular causes, and 292 cases of the expanded composite of major cardiovascular events. The distribution of the study participants by baseline characteristics according to the number of ideal metrics is summarized in Table 1. Participants’ values for systolic and diastolic blood pressure, fasting blood glucose, LDL- and total cholesterol, triglycerides, C-reactive protein, and BMI monotonically decreased as the number of metrics increased, whereas HDL-cholesterol values and MedDiet adherence score gradually increased (Table 1).

**Table 1:** Participant characteristics at baseline according to the number of ideal cardiovascular health in the RIVANA cohort (*n* = 3826)

Baseline characteristics	Number of metrics for ideal cardiovascular health			
	0–2	3	4	5–7
*N* (frequency)	767	1062	948	1049
Age, years	54.8 (11.7)	55.7 (12.3)	53.7 (12.5)	47.7 (10.8)
Sex, men, *n* (%)	513 (66.9%)	538 (50.7%)	400 (42.2%)	238 (22.7%)
Higher level of attained education, *n* (%)				
Primary or less	477 (62%)	662 (62%)	514 (54%)	419 (40%)
Secondary	162 (21%)	217 (20%)	223 (24%)	281 (27%)
College/university	128 (17%)	183 (17%)	211 (22%)	349 (33%)
Occupation, *n* (%)				
Executives/managers	183 (24%)	237 (22%)	242 (26%)	304 (29%)
Clerical workers	225 (29%)	297 (28%)	280 (30%)	296 (28%)
Manual workers	359 (47%)	528 (50%)	426 (45%)	449 (43%)
BMI, kg/m^2^	29.5 (4.2)	28.4 (4.2)	26.4 (4.2)	23.7 (3.1)
Physical activity at baseline, METs-min/week	2028 (2204)	2713 (2263)	2772 (2274)	2960 (2358)
^a^Alcohol consumption, *n* (%)				
Never	364 (47%)	540 (51%)	514 (54%)	648 (62%)
Light	99 (13%)	172 (16%)	160 (17%)	195 (19%)
Moderate	279 (36%)	335 (32%)	262 (28%)	200 (19%)
Heavy	25 (3%)	15 (1%)	12 (1%)	6 (1%)
Adherence to the MedDiet (14 item MedDiet score)	7.6 (1.8)	8.7 (2.1)	9.0 (2.0)	9.3 (1.9)
Systolic BP, mmHg	141 (16)	138 (18)	132 (18)	118 (15)
Diastolic BP, mmHg	84 (9)	83 (10)	80 (10)	73 (9)
Fasting blood glucose, mg/dl	111 (26)	105 (23)	97 (18)	91 (10)
Total cholesterol, mg/dl	227 (35)	221 (36)	210 (36)	198 (37)
LDL cholesterol, mg/dl	141 (33)	136 (33)	125 (31)	113 (32)
HDL cholesterol, mg/dl	58 (15)	62 (16)	65 (16)	71 (16)
Ratio TC: HDL-c (×100)	4.1 (1.2)	3.8 (1.1)	3.4 (0.9)	2.9 (0.7)
Triglycerides, mg/dl	153 (124)	124 (76)	103 (58)	78 (38)
C-reactive protein, mg/dl	10.9 (25.7)	10.1 (24.2)	9.6 (39.0)	5.8 (15.8)
*Medications at baseline, n (%)*				
Antihypertensive therapy	177 (23%)	239 (23%)	142 (15%)	47 (4%)
Lipid-lowering therapy	107 (14%)	119 (11%)	65 (7%)	28 (3%)
Antidiabetic agents	52 (7%)	52 (5%)	22 (2%)	8 (1%)
*Cardiovascular health metrics, n (%)*				
Never-smoker^b^	273 (36%)	704 (66%)	653 (69%)	819 (78%)
MedDiet, ≥ 9 points	178 (23%)	581 (55%)	602 (64%)	788 (75%)
Physical activity, ≥ 500 METs-min/week	572 (75%)	971 (91%)	897 (95%)	1026 (98%)
BMI, < 25 kg/m^2^	41 (5%)	160 (15%)	381 (40%)	811 (77%)
Total cholesterol, untreated and < 200 mg/dl	80 (10%)	210 (20%)	354 (37%)	643 (61%)
Systolic and diastolic BP, untreated and < 120/80 mmHg	18 (2%)	93 (9%)	215 (23%)	686 (65%)
Fasting blood glucose, untreated and < 100 mg/dl	181 (24%)	467 (44%)	690 (73%)	965 (92%)

Values are means (SD) unless otherwise indicated

*Abbreviations*: *BMI* body mass index, *BP* blood pressure, *HDL* high-density lipoprotein, *kg/m*^*2*^ kilograms per (meter squared), *LDL* low-density lipoprotein, *MedDiet* Mediterranean diet, *MET* metabolic equivalent, *min/w* minutes per week, *mg/dL* milligrams per deciliter, *mm/Hg* millimeters of mercury, *Ratio TC* HDL-c ratio total cholesterol to HDL cholesterol

^a^Alcohol consumption was defined as follows: never (0 g/day), light (< 5 g/day), moderate (women: 5–15 g/day and men 5–30 g/day), heavy (women: > 15 g/day and men > 30 g/day)

^b^Never-smoker or quitting > 12 months ago

When examining the distribution of participants according to the number of achieved metrics (Additional file 1: Fig. S2), the highest proportion of participants met 3 metrics (27.8%) or 4 metrics (24.8%), whereas the lowest proportion met 0 metrics (0.3%) or 7 metrics (2.1%). The distribution of participants by each health metric is presented in Additional file 1: Fig. S3. Among our participants, the physical activity metric was met by 90.6% of them, the smoking metric by 64.0%, the fasting blood glucose metric by 60.2%, the healthy diet metric by 56.2%, the blood pressure metric by 26.4%, the total cholesterol metric by 33.6%, and the BMI metric by 36.4%.

**Fig. 1:**
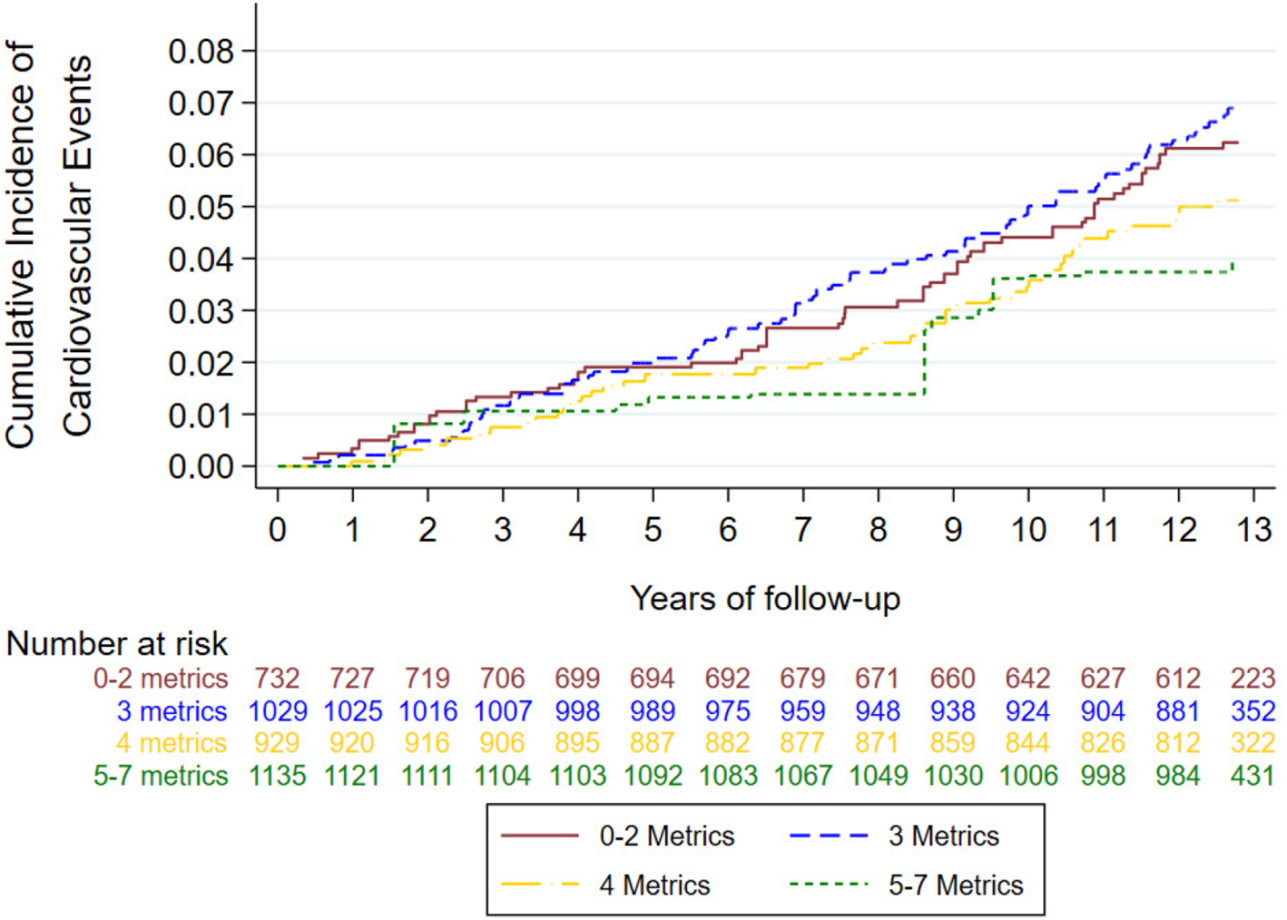
Nelson-Aalen survival plot for the cumulative risk of experiencing major cardiovascular risk events (composite of myocardial infraction, stroke, or death from cardiovascular causes) adjusted through inverse probability weighting according to the categories of ideal cardiovascular health in the RIVANA cohort (*n* = 3826). Models were adjusted for age (continuous), sex, higher level of attained education (primary or less, secondary, and college/university), and occupation (executives/managers, clerical workers, and manual workers)

We represented the cumulative incidence of major cardiovascular events according to ICVH score categories using inverse probability weighting (Fig. 1). Participants meeting a higher ICVH score showed a lower cumulative incidence of cardiovascular major events (log-rank *p* < 0.05).

Estimates of HRs and RAPs for cardiovascular endpoints according to categories of cumulative achieved metrics are shown in Table 2. After multiple-adjustment, participants in the healthiest category (5–7 metrics) compared to participants in the reference group (0–2 metrics) showed a 68% significant relatively lower risk of major cardiovascular events with HR of 0.32 (95% CI 0.17–0.60) and RAP of − 14.36 (95% CI − 22.85, − 5.87). For secondary endpoints, participants in the healthiest category compared to those in the reference category showed a significant relatively lower risk of 68%, 71%, 81%, and 69% for myocardial infarction, stroke, death from cardiovascular causes, and the expanded composite of major cardiovascular events, respectively. The RAPs for participants who achieved ≥ 5 metrics compared with those ≤ 2 metrics were − 27.8 years for myocardial infarction, − 12.8 years for stroke, − 13.8 years for cardiovascular death, and − 17.2 years for the expanded composite of major cardiovascular events. For each additional metric, the risk of major cardiovascular events relatively decreased by 24% (HR of 0.76, 95% CI 0.66–0.88) in the fully adjusted model. When we merged participants in the 2 healthiest score groups into one group and compared them to the reference group (4–7 metrics vs. 0–2 metrics), the inverse association was slightly attenuated for all the outcomes but remained significant for major cardiovascular events, myocardial infarction, and the expanded composite of major cardiovascular events (Additional file 2: Table S1). We evaluated the non-linear relationship between the number of ICVH metrics (continuous) and the primary endpoint (Additional file 1: Fig. S4) and found an inverse significant non-linear relationship (*P*_non-linearity_ = 0.014). The risk of major cardiovascular risk seemed to decrease for participants who achieved > 3 metrics and became significant for those participants who achieved > 4 metrics.

**Table 2:** Estimates of cardiovascular risk according to the number of ideal cardiovascular health metrics in the RIVANA cohort (*n* = 3826)

Endpoint	Number of metrics for ideal cardiovascular health, HR (95% CI)				***P***-trend^***1***^
	0–2 (*n* = 767)	3 (*n* = 1062)	4 (*n* = 948)	5–7 (*n* = 1049)	
**Major cardiovascular disease**^**a**^					
Events/person-years	53/9126	83/12594	46/11482	12/13156	
Incidence rate/10,000 person years	58.07	65.90	40.06	9.12	
Unadjusted model	1.00 (ref.)	1.14 (0.81–1.61)	0.69 (0.46–1.02)	0.16 (0.08–0.29)	< 0.001
Sex- and age-adjusted model	1.00 (ref.)	1.12 (0.79–1.58)	0.78 (0.52–1.16)	0.31 (0.16–0.58)	< 0.001
MV-adjusted model^b^	1.00 (ref.)	1.12 (0.79–1.59)	0.79 (0.53–1.18)	**0.32 (0.17–0.60)**	**< 0.001**
*Rate advancement periods (years)*					
MV-adjusted model^b^	0.00 (ref.)	1.44 (− 2.92, 5.80)	− 2.94 (− 7.94, 2.06)	**− 14.36 (− 22.85, − 5.87)**	
**Myocardial infarction**					
Events/person-years	20/9213	31/12739	11/11579	5/13175	
Incidence rate/10,000 person years	21.71	24.33	9.50	3.80	
Unadjusted model	1.00 (ref.)	1.12 (0.64–1.97)	0.44 (0.21–0.91)	0.18 (0.07–0.47)	< 0.001
Sex- and age-adjusted model	1.00 (ref.)	1.18 (0.67–2.08)	0.52 (0.25–1.09)	0.31 (0.11–0.84)	0.004
MV-adjusted model^b^	1.00 (ref.)	1.18 (0.67–2.08)	0.53 (0.25–1.10)	**0.32 (0.12–0.87)**	**0.006**
*Rate advancement periods (years)*					
MV-adjusted model^b^	0.00 (ref.)	3.74 (− 9.24, 16.73)	− 14.49 (− 32.66, 3.67)	**− 27.79 (− 54.93, − 1.34)**	
**Stroke**					
Events/person-years	23/9216	39/12751	20/11545	5/13184	
Incidence rate/10,000 person years	24.96.	30.59	17.32	37.92	
Unadjusted model	1.00 (ref.)	1.23 (0.73–2.06)	0.69 (0.38–1.26)	0.15 (0.06–0.40)	< 0.001
Sex- and age-adjusted model	1.00 (ref.)	1.17 (0.69–1.96)	0.77 (0.42–1.40)	0.30 (0.11–0.80)	0.011
MV-adjusted model^b^	1.00 (ref.)	1.15 (0.69–1.94)	0.76 (0.41–1.39)	**0.29 (0.11–0.77)**	**0.009**
*Rate advancement periods (years)*					
MV-adjusted model^b^	0.00 (ref.)	1.47 (− 3.92, 6.86)	− 2.87 (− 9.13, 3.39)	**− 12.77 (− 23.44, − 2.10)**	
**Death from cardiovascular causes**					
Events/person-years	17/9313	27/12907	20/11642	2/13204	
Incidence rate/10,000 person years	18.26	20.92	17.18	1.51	
Unadjusted model	1.00 (ref.)	1.15 (0.63–2.11)	0.94 (0.49–1.79)	0.08 (0.02–0.36)	< 0.001
Sex- and age-adjusted model	1.00 (ref.)	1.03 (0.56–1.90)	1.02 (0.53–1.95)	0.18 (0.04–0.77)	0.059
MV-adjusted model^b^	1.00 (ref.)	1.04 (0.57–1.92)	1.03 (0.54–1.97)	**0.19 (0.04–0.80)**	0.072
*Rate advancement periods (years)*					
MV-adjusted model^b^	0.00 (ref.)	0.34 (− 4.63, 5.31)	0.25 (− 5.05, 5.55)	**− 13.77 (− 26.23, − 1.,31)**	
**Expanded composite major cardiovascular disease**^**c**^					
Events/person-years	94/8868	112/12409	64/11372	22/13084	
Incidence rate/10,000 person years	106.00	90.25	56.28	16.81	
Unadjusted model	1.00 (ref.)	0.85 (0.65–1.12)	0.53 (0.39–0.73)	0.16 (0.10–0.25)	< 0.001
Sex- and age-adjusted model	1.00 (ref.)	0.85 (0.64–1.12)	0.61 (0.44–0.84)	0.30 (0.19–0.49)	< 0.001
MV-adjusted model^b^	1.00 (ref.)	0.85 (0.65–1.12)	**0.61 (0.44–0.84)**	**0.31 (0.19–0.49)**	**< 0.001**
*Rate advancement periods (years)*					
MV-adjusted model^b^	0.00 (ref.)	− 2.33 (− 6.36, 1.69)	**− 7.16 (− 11.94, − 2.37)**	**− 17.22 (− 24.84, − 9.59)**	

Bold values are statistically significant at *P* < 0.05

*Abbreviations*: *CI* confidence interval, *HR* hazard ratio, *M-V* multivariate, *Ref*. reference

^1^*P*-value for linear trend

^a^Major cardiovascular disease was defined as occurrence of myocardial infarction, stroke, or cardiovascular death

^b^Multivariate model adjusted for age (continuous), sex, higher level of attained education (primary or less, secondary, and college/university), and occupation (executives/managers, clerical workers, and manual workers)

^c^Expanded composite major cardiovascular disease included myocardial infarction, stroke, cardiovascular death, other ischemic heart diseases, other cerebrovascular diseases, and peripheral arterial disease (Supplemental Fig. S1)

**Fig. 2:**
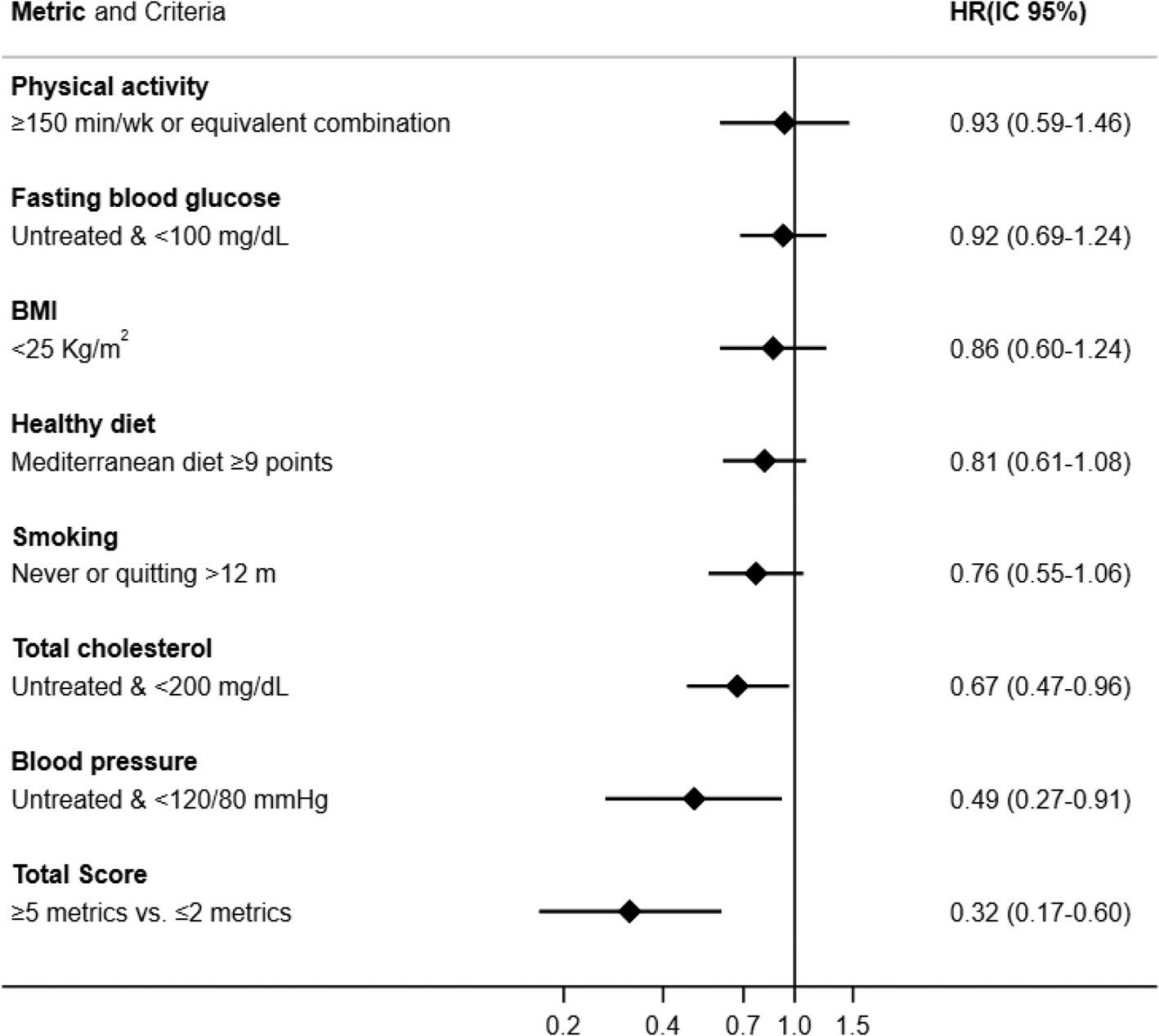
Individual association of each individual metric and their combination. HRs and 95% CIs associated with each of the seven ideal metrics and their combination for the risk of major cardiovascular events in the RIVANA cohort (*n* = 3826). Models were adjusted for age (continuous), sex, higher level of attained education (primary or less, secondary, and college/university), and occupation (executives/managers, clerical workers, and manual workers). Additionally, when an individual metric of the ICVH score was the exposure of interest, the model was mutually adjusted for the other metrics of the ICVH score. Abbreviations: BMI, body mass index; kg/m^2^, kilograms per (meter squared); mg/dL milligrams per deciliter; min/w, minutes per week; mo, months; mm/Hg, millimeters of mercury

The association between individual metrics of the ICVH score and the risk of major cardiovascular events is displayed in Fig. 2. Despite all the metrics suggested an inverse relationship, only total cholesterol (HR 0.67, 95% CI 0.47–0.96) and blood pressure (HR 0.49, 95% CI 0.27–0.91) showed a significant inverse relationship with major cardiovascular events, in the models which were mutually adjusted for all other metrics. The greatest benefit in terms of reducing risk of cardiovascular events was suggested by combining ≥ 5 metrics. The limited number of major cardiovascular events in our study might have precluded from finding significant associations for the individual metrics.

When we performed sensitivity analyses with additional adjustments for alcohol consumption and serum concentration of high-sensitivity C-reactive protein (continuous), results barely changed (Additional file 2: Table S2). Additionally, the results of the sensitivity analysis for the association of individual metrics with the risk of major cardiovascular events (removing from the models the effect of the other metrics) did not reveal any major change (Additional file 1: Fig. S5).

## Discussion

We aimed to prospectively examine the association of ICVH metrics and cardiovascular events in the RIVANA cohort, a population-based cohort of approximately 4000 participants. Our findings suggested a strong inverse association between a higher number of ideal metrics and major cardiovascular events (composite of myocardial infarction, stroke, or death from cardiovascular causes). Higher number of ideal metrics was also associated with a significantly lower risk of infarction, stroke, cardiovascular death, and an expanded composite of major cardiovascular events. The relative risk reduction for participants in the healthiest category (5–7 metrics) compared to participants in the unhealthiest (0–2 metrics) was 68% for risk of major cardiovascular risk events, and 68%, 71%, 81%, and 69% for myocardial infarction, stroke, cardiovascular death, and the expanded composite of major cardiovascular events, respectively. Regarding the magnitude of the RAP, we found that participants who met at least 5 metrics showed the same risk of cardiovascular major events as those participants with 2 or fewer metrics approximately 18 years later; these age difference resulted in 28 years for myocardial infarction, 13 years for stroke, 14 years for cardiovascular death, and 17 years for the expanded composite of major cardiovascular events.

In our population-based cohort, only 2.1% of the participants achieved ICVH, that is, met all 7 health metrics. The proportion rose up to 8.8% for 6 health metrics and 16.6% for 5 health metrics. These results are consistent with previous US and non-US studies [9], in which approximately < 20% of adults met at least 5 metrics, suggesting that adherence to healthy cardiovascular habits is a global concern. At the national level, the 27.5% of participants of our study that met ≥ 5 metrics was higher than the 18.9% found in a previous study conducted in the ENRICA cohort [41], a representative sample of noninstitutionalized adult population of Spain, and considerably higher than the 3.5% observed in the participants of the PREDIMED trial [19], which may be explained by the inclusion criteria of the PREDIMED study (advanced age adults at high cardiovascular risk). Noteworthy, a large proportion of participants of our study met the metric for physical activity, which is consistent with the fact that inhabitants of the Autonomous Community of Navarre show one of the highest levels of physical activity throughout Spain [42]. Observing the large proportion of participants who met the metric for physical activity and healthy diet (56.2%), a higher proportion of participants achieving the metrics for blood pressure and total cholesterol might be expected. Potential explanations for these findings may rely on reverse causality—participants may improve their quality diet and physical activity levels in response to the diagnosis of some risk factors such as elevated blood pressure or elevated cholesterol levels.

Our findings are in line with previous studies conducted in the last few years across different countries that have suggested the health benefits of adhering to ICVH metrics [9, 43]. Significant reductions in cardiovascular risk have been reported as individuals meet a higher number of ideal metrics, observing a relative risk reduction greater than 60% in the majority of studies for adults who meet 6 or 7 metrics compared to individuals with 0 to 2 metrics. In Spain, only the aforementioned study conducted in the PREDIMED trial has examined the association of the 7 ideal metrics with cardiovascular risk [19]. Our findings support that the risk of major cardiovascular events decreased when participants achieved > 3 metrics, observing a significant relative risk reduction when participants achieved > 4 metrics. Despite in our study the relative risk reduction was smaller than the one observed in the PREDIMED (39% *vs.* 64% when compared participants who achieved ≥ 4 metrics with participants in the lowest category), our findings additionally support the benefits of adhering to ideal metrics for the general population at low or medium cardiovascular risk. Furthermore, around 50% of the adult population of our cohort did not meet 4 health metrics, which highlights the potential impact of health promotion programs aimed to improve cardiovascular health behaviors and factors.

In our study, we additionally observed an inverse association between the risk of major cardiovascular events and individual metrics of the score. None of the individual metrics, however, showed a greater protective effect than their total combination. These findings highlight the need to reinforce a direct message to the population about the importance of cardiovascular prevention from a multidimensional approach. Adhering to as many healthy cardiovascular habits as possible, instead of focusing on just one or of a few metrics, should be the public health message to deliver.

In the context of risk communication, RAPs may represent a more comprehensible approach than classical risk estimates for the general population to understand the impact of the benefits of adhering to ideal metrics. Individuals generally expect to be informed about certain risk in a simple and understandable manner so that they can decide whether to change their behavior [44]. Moreover, health messages need to be relevant to the population at risk to capture their attention and increase risk perception. RAP represents the risk associated with ICVH in respect to the timing of occurrence of cardiovascular events. As such, our findings estimated that a 60-year-old adult who met ≤ 2 health metrics would experience the same risk of major cardiovascular events as a 74-year-old adult who met ≥ 5 metrics. This *advancement* (years of excess risk) may result in a simpler and more straightforward message to the general population. When individuals understand the risk of certain health factors and the potential benefits or prevention, shared decision-making may be more likely to occur and may consequently lead to behavioral change [10].

### Limitations and strengths

This study has several limitations. First, ICVH metric information was assessed at baseline, and it may be possible that participants might have changed their metrics over follow-up. Second, despite our statistical models were adjusted for potential confounders, the absence of confounding cannot be completely ruled out. Third, the number of study endpoints in our cohort was limited, particularly secondary endpoints, which may have attenuated the statistical power of our results; nevertheless, previous findings of the RIVANA cohort on CVD have been reliable [23]. And fourth, it may be possible that certain lifestyle behaviors such as physical activity, diet, or smoking may be greatly rooted in a country’s culture; yet, it seems plausible the observed findings can be generalized to other populations. Despite these limitations, the strengths of our study are reflected in the population-based nature of the cohort, the rigorous method of collection of cardiovascular events supported by public resources and validated electronic health record database [36, 37], the long follow-up, the high retention proportion, and the inclusion of sensitivity analyses.

## Conclusions

The results of our study suggest a substantially lower risk of major cardiovascular events, myocardial infarction, stroke, cardiovascular death, and the expanded composite of major cardiovascular events when adhering to ICVH. RAPs demonstrated the potential for prevention by adhering to health metrics. We additionally found that the combined effects of the ICVH metrics led to a lower risk reduction than the individual effect of any metric. Public health efforts should take a multidimensional approach for health promotion to reduce the CVD epidemic.

## Supplementary Information

**Additional file 1: Figure S1.** Diagnostic criteria for endpoints of the study. **Figure S2.** Distribution of participants in the RIVANA cohort (*n*=3,826) according to the number of achieved metrics. **Figure S3.** Distribution of participants in the RIVANA cohort by each health metric (*n*=3,826). **Figure S4.** Restricted Cubic Splines for the Hazard Ratio (HR) and 95% Confidence Interval (CI) for the number of ICVH metrics and major cardiovascular events in the RIVANA cohort (*n* = 3,826). **Figure S5.** Individual association of each individual metric and their combination. HRs and 95% CIs associated with each of the seven ideal metrics and their combination for the risk of major cardiovascular events in the RIVANA cohort (*n*=3,826).

**Additional file 2: Table S1.** Estimates of Cardiovascular Risk According to the Number of Ideal Cardiovascular Health (3 groups: 0-2, 3, and 4-7 metrics) in the RIVANA cohort (*n*= 3,826). **Table S2.** Sensitivity Analysis. Estimates of Cardiovascular Events According to the Number of Ideal Cardiovascular Health in the RIVANA cohort (*n*= 3,826).

## Data Availability

Data will be available upon reasonable request from the corresponding author.

## Abbreviations

AHA: American Heart Association
CI: Confidence interval
CVD: Cardiovascular disease
HR: Hazard ratio
ICVH: Ideal cardiovascular health
MedDiet: Mediterranean diet
METs: Metabolic equivalents
RAP: Rate advancement period
RIVANA: Vascular Risk in Navarra
SD: Standard deviation
