# Supplementary material for: Association of ideal cardiovascular health with cardiovascular events and risk advancement periods in a Mediterranean population-based cohort
Source: BMC Med. 2022 Jul 5;20:232. doi: 10.1186/s12916-022-02417-x (PMC9254604; doi:10.1186/s12916-022-02417-x)
Supplement: Supplementary file 1 — Additional file 1: Figure S1. Diagnostic criteria for endpoints of the study. Figure S2. Distribution of participants in the RIVANA cohort (n=3,826) according to the number of achieved metrics. Figure S3. Distribution of participants in the RIVANA cohort by each health metric (n=3,826). Figure S4. Restricted Cubic Splines for the Hazard Ratio (HR) and 95% Confidence Interval (CI) for the number of ICVH metrics and major cardiovascular events in the RIVANA cohort (n = 3,826). Figure S5. Individual association of each individual metric and their combination. HRs and 95% CIs associated with each of the seven ideal metrics and their combination for the risk of major cardiovascular events in the RIVANA cohort (n=3,826). [file 12916_2022_2417_MOESM1_ESM.docx]

**ADDITIONAL FILE 1**

**Association of Ideal Cardiovascular Health with Cardiovascular Events and Risk Advancement Periods in a Mediterranean Population-Based Cohort**

**Cesar I. Fernandez-Lazaro et al.**

**FIGURES**

**Supplementary Figure s1.** Diagnostic criteria for endpoints of the study.

**Supplementary Figure s2.** Distribution of participants in the RIVANA cohort according to the number of achieved metrics (n=3,826).

**Supplementary Figure s3.** Distribution of participants in the RIVANA cohort by each health metric (n=3,826).

**Supplementary Figure s4.** Restricted Cubic Splines for the Hazard Ratio (HR) and 95% Confidence Interval (CI) for the number of ICVH metrics and major cardiovascular events in the RIVANA cohort (n = 3,826). Solid black line represents the HR and the dashed lines represent the 95% CI. Likelihood ratio test was used to test for non-linearity (*P*_non-linearity_ = 0.014). The model was adjusted for age (continuous), sex, higher level of attained education (primary or less, secondary, and college/university), and occupation (executives/managers, clerical workers, and manual workers).

**Supplementary Figure s5**. Individual association of each individual metric and their combination. HRs and 95% CIs associated with each of the seven ideal metrics and their combination for the risk of major cardiovascular events in the RIVANA cohort (n= 3,826).

**FIGURES**

1. **Primary endpoint**

The primary endpoint of the study was a composite of myocardial infarction, stroke, and death from cardiovascular causes (all major CVD events) and were defined according to the International Classification of Diseases, Ninth and Tenth Revision (ICD-9 and ICD-10) and satisfying the following criteria:

**A) Myocardial infarction (CIE-9: 410-411; CIE-10: I21-I22)**

Criteria: One of the following criteria satisfies the diagnosis of acute myocardial infraction

- Presence of ECG (Development of pathologic Q waves in the ECG throughout the acute phase) or presence of troponins (>percentile 95) with clinical suggestion of myocardial infraction, and in the following cases:
  - - Common, uncommon or poorly described symptoms, along with probable ECG (modification of the ST segment or inverted T wave during the episode) and abnormal enzymes (CK>percentile 95 or CK-MB>percentile 99) in the absence of troponins
- Common symptoms, abnormal enzymes (CK>percentile 95 or CK-MB>percentile 99) and ischemic ECG (modification of the ST segment or inverted T wave during episode), non-codable (due to branch blockage or presence of pacemaker), or not available.
- Diagnosis from a pathological examination

**B) Stroke (CIE-9: 430-438 CIE-10: I60-I69)**

Acute neurological deficit lasting more than 24 hours caused by focal ischemia cerebral caused by the following syndromes: lacunar infarction, atherothrombotic infarction, infarction of cardio-embolic origin (secondary to arrhythmias, valvular diseases, or cardiopathies embolization), cerebral and subarachnoid hemorrhages. We excluded those secondary to hematological diseases (leukemias, polycythemia vera), brain metastases and head injuries. The diagnosis must be confirmed by an imaging test (CT or MRI) or confirmed by a pathological examination.

**C) Death from cardiovascular causes**

We included the following cardiovascular causes according to the International Classification of Diseases, Ninth and Tenth Revision (ICD-9 and ICD-10):

- CIE-9: 401-405, 410-414, 426-428, 429.1-429.9, 430-435, 436-438 (excluding 437.4-437.8), 443.8, 443.9, 798.1; 798.2
- CIE-10: G45-G46, I05-I09, I10-I16, I20-I25, I26-I52, I60-I69, I70-I79, I99, R00-R01

1. **Secondary endpoints**

The secondary endpoints of the study included the individual components of the main composite, death from any cause, and an expanded composite of major cardiovascular outcome that additionally included other ischemic heart diseases, other cerebrovascular diseases, and peripheral arterial:

**A) Myocardial infarction** (defined above)

**B) Stroke** (defined above)

**C) Death from cardiovascular causes** (defined above)

**D) Expanded composite major cardiovascular outcome**

Defined according to the ICD-9 and ICD-10:

- CIE-9: 410.*-414.*, 430.*-438.*, 443.* and death from 401-405, 410-414, 426-428, 429.1-429.9, 430-435, 436-438 (excluding 437.4-437.8), 443.8, 443.9, 798.1; 798.2
- CIE-10: G45.*, G46.*, I20.0-I25.9, I60.0-I69.9, I73.*, I79.* and death from G45-G46, I05-I09, I10-I16, I20-I25, I26-I52, I60-I69, I70-I79, I99, R00-R01

**Supplementary Figure s1.** Diagnostic criteria for endpoints of the study.


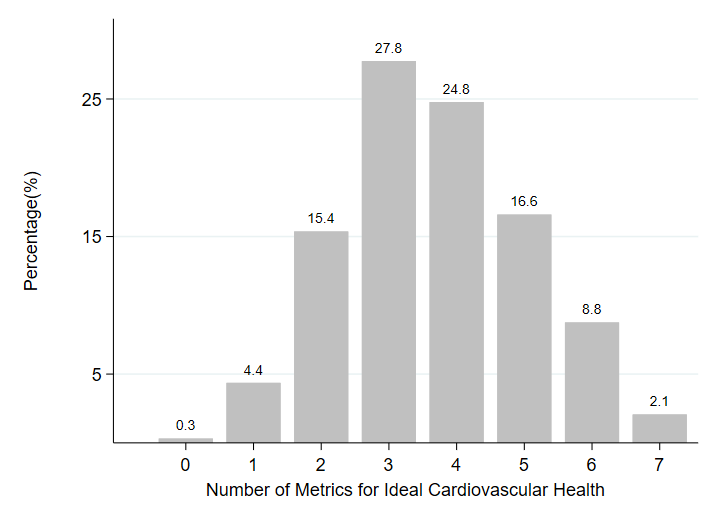


**Supplementary Figure s2.** Distribution of participants in the RIVANA cohort according to the number of achieved metrics (n=3,826).


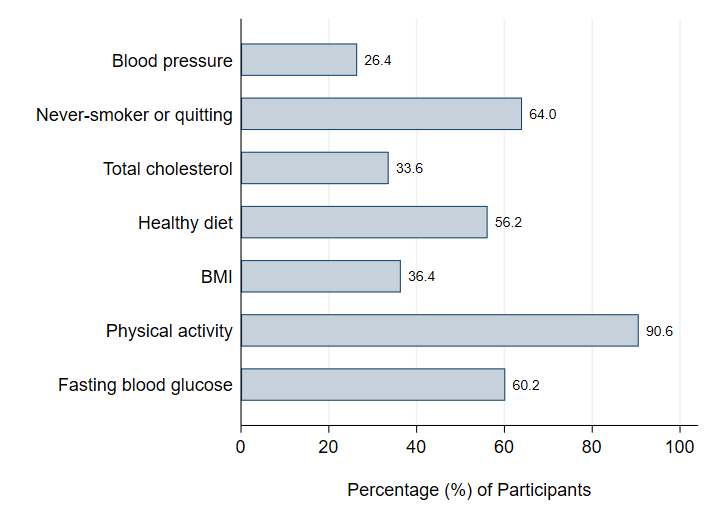


**Supplementary Figure s3.** Distribution of participants in the RIVANA cohort by each health metric (n=3,826).


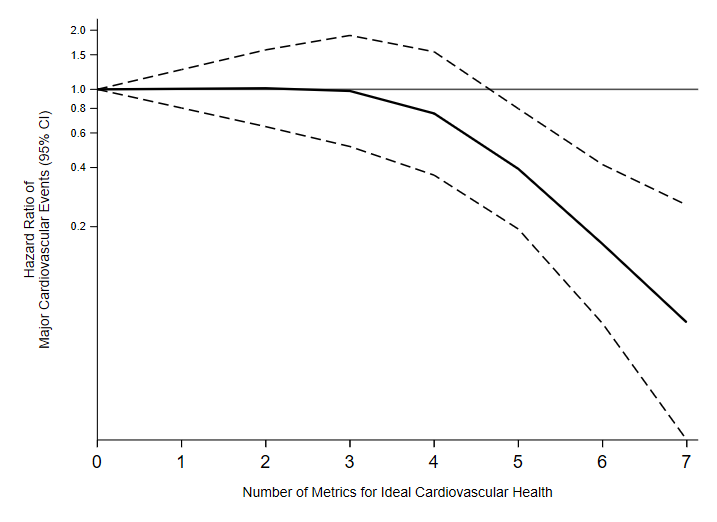


*P _non-linearity_* = 0.014

**Supplementary Figure s4.** Restricted Cubic Splines for the Hazard Ratio (HR) and 95% Confidence Interval (CI) for the number of ICVH metrics and major cardiovascular events in the RIVANA cohort (n = 3,826). Solid black line represents the HR and the pointed lines represent the 95% CI. Likelihood ratio test was used to test for non-linearity (*P*_non-linearity_ = 0.014). The model included was adjusted for age (continuous), sex, higher level of attained education (primary or less, secondary, and college/university), and occupation (executives/managers, clerical workers, and manual workers).


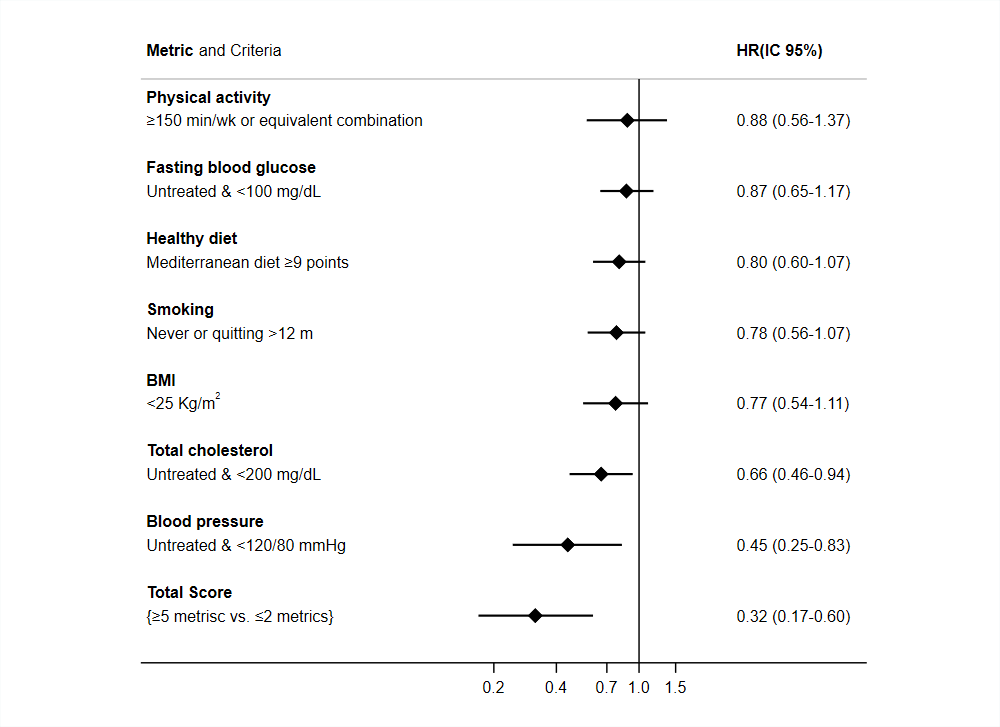


**Supplementary Figure s5.** Individual association of each individual metric and their combination. HRs and 95% CIs associated with each of the seven ideal metrics and their combination for the risk of major cardiovascular events in the RIVANA cohort (n=3,826).

Abbreviations: BMI, body mass index; kg/m2, kilograms per (meter squared); mg/dL milligrams per deciliter; min/w, minutes per week; mo, months; mm/Hg, millimeters of mercury.
Models were adjusted for age (continuous), sex, higher level of attained education (primary or less, secondary, and college/university), and occupation (executives/managers, clerical workers, and manual workers).
