# Supplementary material for: Association of ideal cardiovascular health with cardiovascular events and risk advancement periods in a Mediterranean population-based cohort
Source: BMC Med. 2022 Jul 5;20:232. doi: 10.1186/s12916-022-02417-x (PMC9254604; doi:10.1186/s12916-022-02417-x)
Supplement: Supplementary file 2 — Additional file 2: Table S1. Estimates of Cardiovascular Risk According to the Number of Ideal Cardiovascular Health (3 groups: 0-2, 3, and 4-7 metrics) in the RIVANA cohort (n= 3,826). Table S2. Sensitivity Analysis. Estimates of Cardiovascular Events According to the Number of Ideal Cardiovascular Health in the RIVANA cohort (n= 3,826). [file 12916_2022_2417_MOESM2_ESM.docx]

**ADDITIONAL FILE 2**

**Association of Ideal Cardiovascular Health with Cardiovascular Events and Risk Advancement Periods in a Mediterranean Population-Based Cohort**

**Cesar I. Fernandez-Lazaro et al.**

**TABLES**

**Supplementary Table s1.** Estimates of Cardiovascular Risk According to the Number of Ideal Cardiovascular Health (3 groups: 0-2, 3, and 4-7 metrics) in the RIVANA cohort (n= 3,826).

**Supplementary Table s2.** Sensitivity Analysis. Estimates of Cardiovascular Events According to the Number of Ideal Cardiovascular Health in the RIVANA cohort (n= 3,826).

| **Supplementary Table s1.** Estimates of Cardiovascular Risk According to the Number of Ideal Cardiovascular Health (3 groups: 0-2, 3, and 4-7 metrics) in the RIVANA cohort (n= 3,826). | | | | |
| --- | --- | --- | --- | --- |
|  | **Number of Metrics for Ideal Cardiovascular Health, HR (95% CI)** | | | |
| **Endpoint** | 0-2  (n = 767) | 3 (n = 1,062) | 4-7 (n = 1,997) | ***p*-trend*^1^*** |
|  |  |  |  |  |
| **Major cardiovascular disease^2^** |  |  |  |  |
| Events/Person-years | 53/9126 | 83/12594 | 58/24637 |  |
| Incidence rate/10,000 person years | 58.07 | 65.90 | 23.54 |  |
| Unadjusted Model | 1.00 (ref.) | 1.14 (0.81 - 1.61) | 0.40 (0.28 - 0.59) | < 0.001 |
| Sex and Age Adjusted Model | 1.00 (ref.) | 1.12 (0.79 - 1.58) | 0.60 (0.41 - 0.87) | 0.001 |
| MV-Adjusted Model^3^ | 1.00 (ref.) | 1.12 (0.79 - 1.59) | **0.61 (0.42 - 0.89)** | **0.002** |
| *Rate Advancement Periods (years)* |  |  |  |  |
| MV-Adjusted Model^3^ | 0.00 (ref.) | 1.42 (-2.84, 5.68) | **-6.02 (-10.77, -1.27)** |  |
| **Myocardial infarction** |  |  |  |  |
| Events/Person-years | 20/9213 | 31/12739 | 16/24754 |  |
| Incidence rate/10,000 person years | 21.71 | 24.33 | 6.46 |  |
| Unadjusted Model | 1.00 (ref.) | 1.12 (0.64 - 1.97) | 0.30 (0.15 - 0.58) | < 0.001 |
| Sex and Age Adjusted Model | 1.00 (ref.) | 1.18 (0.67 - 2.08) | 0.43 (0.22 - 0.85) | 0.004 |
| MV-Adjusted Model^3^ | 1.00 (ref.) | 1.18 (0.67 - 2.08) | **0.44 (0.22 - 0.87)** | **0.005** |
| *Rate Advancement Periods (years)* |  |  |  |  |
| MV-Adjusted Model^3^ | 0.00 (ref.) | 3.69 (-8.98, 16.36) | **-18.01 (-35.56, -0.46)** |  |
| **Stroke** |  |  |  |  |
| Events/Person-years | 23/9216 | 39/12751 | 25/24729 |  |
| Incidence rate/10,000 person years | 24.96. | 30.59 | 10.11 |  |
| Unadjusted Model | 1.00 (ref.) | 1.23 (0.73 - 2.06) | 0.40 (0.23 - 0.71) | < 0.001 |
| Sex and Age Adjusted Model | 1.00 (ref.) | 1.17 (0.69 - 1.96) | 0.59 (0.33 - 1.05) | 0.021 |
| MV-Adjusted Model^3^ | 1.00 (ref.) | 1.15 (0.69 - 1.94) | 0.58 (0.33 - 1.03) | **0.019** |
| *Rate Advancement Periods (years)* |  |  |  |  |
| MV-Adjusted Model^3^ | 0.00 (ref.) | 1.45 (-3.84, 6.74) | -5.54 (-11.50, 0.43) |  |
| **Death from cardiovascular causes** |  |  |  |  |
| Events/Person-years | 17/9313 | 27/12907 | 22/24846 |  |
| Incidence rate/10,000 person years | 18.26 | 20.92 | 8.85 |  |
| Unadjusted Model | 1.00 (ref.) | 1.15 (0.63 - 2.11) | 0.48 (0.26 - 0.91) | 0.004 |
| Sex and Age Adjusted Model | 1.00 (ref.) | 1.03 (0.56 - 1.90) | 0.71 (0.38 - 1.35) | 0.205 |
| MV-Adjusted Model^3^ | 1.00 (ref.) | 1.04 (0.57 - 1.92) | 0.73 (0.39 - 1.38) | 0.234 |
| *Rate Advancement Periods (years)* |  |  |  |  |
| MV-Adjusted Model^3^ | 0.00 (ref.) | 0.33 (-4,56, 5.22) | -2.52 (-7.66, 2.62) |  |
| **Expanded composite major cardiovascular disease^4^** |  |  |  |  |
| Events/Person-years | 94/8868 | 112/12409 | 86/24456 |  |
| Incidence rate/10,000 person years | 106.00 | 90.25 | 35.17 |  |
| Unadjusted Model | 1.00 (ref.) | 0.85 (0.65 - 1.12) | 0.33 (0.25 - 0.44) | < 0.001 |
| Sex and Age Adjusted Model | 1.00 (ref.) | 0.85 (0.64 - 1.12) | 0.49 (0.36 - 0.66) | < 0.001 |
| MV-Adjusted Model^3^ | 1.00 (ref.) | 0.85 (0.65 - 1.12) | **0.49 (0.37 - 0.66)** | **< 0.001** |
| *Rate Advancement Periods (years)* |  |  |  |  |
| MV-Adjusted Model^3^ | 0.00 (ref.) | -2.26 (-6,19, 1.66) | **-10.07 (-14.63, -5.51)** |  |

Abbreviations: CI, confidence interval; HR, hazard ratio; M-V, multivariate; ref., reference.

Bold values are statistically significant at P<0.05

^1^*P*-value for linear trend

^2^Major cardiovascular disease was defined as occurrence of myocardial infarction, stroke, or cardiovascular death.

^3^Multivariate model adjusted for age (continuous), sex, higher level of attained education (primary or less, secondary, and college/university), and occupation (executives/managers, clerical workers, and manual workers).

^4^Expanded composite major cardiovascular disease included myocardial infarction, stroke, cardiovascular death, other ischemic heart diseases, other cerebrovascular diseases, and peripheral arterial disease (Supplemental Figure s1).

| **Supplementary Table s2.** Sensitivity Analysis. Estimates of Cardiovascular Risk According to the Number of Ideal Cardiovascular Health in the RIVANA cohort (n= 3,826). | | | | | |
| --- | --- | --- | --- | --- | --- |
|  | **Number of Metrics for Ideal Cardiovascular Health, HR (95% CI)** | | | |  |
| **Endpoint** | 0-2 | 3 | 4 | 5-7 | ***p*-trend*^1^*** |
|  |  |  |  |  |  |
| **Major cardiovascular disease^2^** |  |  |  |  |  |
| MV-Adjusted Model^3^ | 1.00 (ref.) | 1.12 (0.79 - 1.59) | 0.79 (0.53 - 1.18) | **0.32 (0.17 - 0.60)** | **< 0.001** |
| MV-Adjusted Model^4^ | 1.00 (ref.) | 1.12 (0.79 - 1.59) | 0.79 (0.53 - 1.18) | **0.32 (0.17 - 0.60)** | **< 0.001** |
| MV-Adjusted Model^5^ | 1.00 (ref.) | 1.12 (0.79 - 1.59) | 0.79 (0.53 - 1.18) | **0.32 (0.17 - 0.60)** | **< 0.001** |
| *Rate Advancement Periods (years)* |  |  |  |  |  |
| MV-Adjusted Model^3^ | 0.00 (ref.) | 1.48 (-2.91, 5.86) | -2.95 (-7.97, 2.07) | **-14.53 (-23.07, -5.99)** |  |
| MV-Adjusted Model^4^ | 0.00 (ref.) | 1.44 (-2.92, 5.81) | -2.96 (-7.96, 2.04) | **-14.30 (-22.79, -5.80)** |  |
| MV-Adjusted Model^5^ | 0.00 (ref.) | 1.48 (-2.91, 5.87) | -2.97 (-8.00, 2.06) | **-14.47 (-23.02, -5.93)** |  |
| **Myocardial infarction** |  |  |  |  |  |
| MV-Adjusted Model^3^ | 1.00 (ref.) | 1.18 (0.67 - 2.08) | 0.52 (0.25 - 1.10) | **0.32 (0.12 - 0.87)** | **0.005** |
| MV-Adjusted Model^4^ | 1.00 (ref.) | 1.18 (0.67 - 2.08) | 0.53 (0.25 - 1.11) | **0.32 (0.12 - 0.88)** | **0.006** |
| MV-Adjusted Model^5^ | 1.00 (ref.) | 1.18 (0.67 - 2.08) | 0.52 (0.25 - 1.10) | **0.32 (0.12 - 0.87)** | **0.005** |
| *Rate Advancement Periods (years)* |  |  |  |  |  |
| MV-Adjusted Model^3^ | 0.00 (ref.) | 3.77 (-9.24, 16.78) | -14.59 (-32.83, 3.64) | **-27.99 (-55.21, -1.22)** |  |
| MV-Adjusted Model^4^ | 0.00 (ref.) | 3.75 (-9.25, 16.75) | -14.51 (-32.71, 3.69) | **-27.75 (-54.93, -1.42)** |  |
| MV-Adjusted Model^5^ | 0.00 (ref.) | 3.77 (-9.26, 16.81) | -14.61 (-32.87, 3.66) | **-27.95 (-55.91, -1.30)** |  |
| **Stroke** |  |  |  |  |  |
| MV-Adjusted Model^3^ | 1.00 (ref.) | 1.16 (0.69 - 1.95) | 0.76 (0.42 - 1.39) | **0.29 (0.11 - 0.77)** | **0.009** |
| MV-Adjusted Model^4^ | 1.00 (ref.) | 1.15 (0.69 - 1.94) | 0.76 (0.41 - 1.38) | **0.29 (0.11 - 0.78)** | **0.009** |
| MV-Adjusted Model^5^ | 1.00 (ref.) | 1.16 (0.69 - 1.95) | 0.76 (0.42 - 1.39) | **0.29 (0.11 - 0.77)** | **0.009** |
| *Rate Advancement Periods (years)* |  |  |  |  |  |
| MV-Adjusted Model^3^ | 0.00 (ref.) | 1.53 (-3.91, 6.98) | -2.84 (-9.16, 3.47) | **-12.97 (-23.74, -2.20)** |  |
| MV-Adjusted Model^4^ | 0.00 (ref.) | 1.48 (-3.92, 6.88) | -2.88 (-9.15, 3.39) | **-12.72 (-23.40, -2.04)** |  |
| MV-Adjusted Model^5^ | 0.00 (ref.) | 1.54 (-3.91, 6.99) | -2.86 (-9.18, 3.47) | **-12.92 (-23.70, -2.14)** |  |
| **Death from cardiovascular causes** |  |  |  |  |  |
| MV-Adjusted Model^3^ | 1.00 (ref.) | 1.04 (0.57 - 1.92) | 1.03 (0.54 - 1.97) | **0.18 (0.04 - 0.80)** | 0.072 |
| MV-Adjusted Model^4^ | 1.00 (ref.) | 1.04 (0.57 - 1.92) | 1.03 (0.54 - 1.97) | **0.19 (0.04 - 0.81)** | 0.075 |
| MV-Adjusted Model^5^ | 1.00 (ref.) | 1.04 (0.57 - 1.91) | 1.03 (0.54 - 1.97) | **0.19 (0.04 - 0.81)** | 0.074 |
| *Rate Advancement Periods (years)* |  |  |  |  |  |
| MV-Adjusted Model^3^ | 0.00 (ref.) | 0.34 (-4.64, 5.32) | 0.26 (-5.05, 5.56) | **-13.80 (-26.29, -1.31)** |  |
| MV-Adjusted Model^4^ | 0.00 (ref.) | 0.34 (-4.64, 5.31) | 0.22 (-5.08, 5.52) | **-13.67 (-26.14, -1.21)** |  |
| MV-Adjusted Model^5^ | 0.00 (ref.) | 0.34 (-4.64, 5.32) | 0.22 (-5.09, 5.53) | **-13.70 (-26.19, -1.21)** |  |
| **Expanded composite major cardiovascular disease^6^** |  |  |  |  |  |
| MV-Adjusted Model^3^ | 1.00 (ref.) | 0.85 (0.65 - 1.12) | **0.61 (0.44 - 0.84)** | **0.31 (0.19 - 0.49)** | **< 0.001** |
| MV-Adjusted Model^4^ | 1.00 (ref.) | 0.85 (0.65 - 1.12) | 0.61 (0.44 - 0.84) | **0.31 (0.19 - 0.49)** | **< 0.001** |
| MV-Adjusted Model^5^ | 1.00 (ref.) | 0.85 (0.65 - 1.12) | 0.61 (0.44 - 0.84) | **0.31 (0.19 - 0.49)** | **< 0.001** |
| *Rate Advancement Periods (years)* |  |  |  |  |  |
| MV-Adjusted Model^3^ | 0.00 (ref.) | -2.32 (-6.36, 1.71) | **-7.16 (-11.96, -2.37)** | **-17.35 (-25.00, -9.69)** |  |
| MV-Adjusted Model^4^ | 0.00 (ref.) | -2.33 (-6.36, 1.69) | **-7.16 (-11.94, -2.37)** | **-17.20 (-24.84, -9.57)** |  |
| MV-Adjusted Model^5^ | 0.00 (ref.) | -2.32 (-6.36, 1.72) | **-7.17 (-11.97, -2.37)** | **-17.34 (-25.00, -9.68)** |  |

Abbreviations: CI, confidence interval; HR, hazard ratio; M-V, multivariate; ref., reference.

Bold values are statistically significant at P<0.05

^1^*P*-value for linear trend

^2^Major cardiovascular disease was defined as occurrence of myocardial infarction, stroke, or cardiovascular death.

^3^Multivariate model adjusted for age (continuous), sex, higher level of attained education (primary or less, secondary, and college/university), occupation (executives/managers, clerical workers, and manual workers), and additionally adjusted for alcohol consumption (never [0 g/day]; light [<5 g/day]; moderate [women: 5-15 g/day & men 5-30 g/day]; heavy [women: >15 g/day & men >30 g/day]).

^4^Multivariate model adjusted for age (continuous), sex, higher level of attained education (primary or less, secondary, and college/university), occupation (executives/managers, clerical workers, and manual workers), and additionally adjusted for serum concentration of high-sensitivity C-reactive protein (continuous).

^5^Multivariate model adjusted for age (continuous), sex, higher level of attained education (primary or less, secondary, and college/university), occupation (executives/managers, clerical workers, and manual workers), and additionally adjusted for alcohol consumption (never [0 g/day]; light [<5 g/day]; moderate [women: 5-15 g/day & men 5-30 g/day]; heavy [women: >15 g/day & men >30 g/day]) and serum concentration of high-sensitivity C-reactive protein (continuous).

^6^Expanded composite major cardiovascular disease included myocardial infarction, stroke, cardiovascular death, other ischemic heart diseases, other cerebrovascular diseases, and peripheral arterial disease (Additional file 1: Figure s1).
